# Renal dysfunction as a predictor of acute stroke outcomes

**DOI:** 10.17712/nsj.2017.4.20170185

**Published:** 2017-10

**Authors:** Danah K. AboAlSamh, Ahmad A. Abulaban, Ismail A. Khatri, Ali M. Al-Khathaami

**Affiliations:** *From the Department of Medicine (Abulaban, Khatri, Al-Khathaami), King Abdulaziz Medical City, from King Abdullah International Medical Research Center (AboAlSamh, Al-Khathaami), from King Saud Bin AbdulAziz University for Health Sciences (Khatri), National Guard Health Affairs, Riyadh, Kingdom of Saudi Arabia*

## Abstract

**Objectives:**

To explore if renal dysfunction in terms of estimated glomerular filtration rate (eGFR) can be considered a risk factor for stroke outcomes.

**Methods:**

The study population consisted of adults diagnosed with acute stroke admitted to the King Abdulaziz Medical City, Riyadh, Kingdom of Saudi Arabia between 2012 and 2015. Data was collected by chart review. The Modification of Diet in Renal Disease equation was used to estimate GFR. Patients were classified into 2 eGFR categories: eGFR >60 (normal) and eGFR ≤60 (low).

**Results:**

A total of 727 patients were studied of whom 596 (82%) had normal eGFR and 131 (18%) had low eGFR. There were more males (68.5%). Ischemic strokes were more prevalent (87.2%). Urinary tract infections were more likely to occur in the low eGFR group (OR=2.047, 95% CI=1.024 – 4.093). They were also significantly more likely to die during admission (OR=3.772, 95% CI=1.609 – 8.844). There was a statistically significant degree of disability reflected by higher mRS (p=0.010) as well as higher post-stroke National Institute of Health Stroke Score scores in the low eGFR group (p=0.011).

**Conclusion:**

Estimated glomerular filtration rate is a possible predictor of stroke severity, disability and mortality.

The relationship between kidney dysfunction and ischemic heart disease (IHD) has been extensively researched in the past 2 decades. This association is thought to be due to the metabolic, endocrine and volume abnormalities that develop when kidneys fail.^1^ The uremia of chronic kidney disease (CKD) has also been implicated in atherosclerotic plaque development.^2^ Although acute stroke shares the risk factors associated with IHD, the relationship between renal dysfunction and stroke is difficult to elucidate. An association is particularly difficult to elicit in the early stages of CKD.^3^

Most studies agree that End Stage Renal Disease patients are at a higher risk for stroke compared to the general population.^4^ Results are less consistent in patients with earlier stages of CKD. The outcomes of stroke in patients with renal impairment have also not been fully explored in comparison to patients with normal kidney functions. In a recent paper, a study conducted on Chinese patients documented higher mortality in diabetic acute stroke patients with impaired kidney function.^5^

Stroke patients represent a massive burden on any health care system and Saudi Arabia is no exception. The most recent study concerning prevalence in Saudi Arabia was conducted in 1993 by Al Rajeh et al and found a prevalence of 43.8 cases per 100,000.^6^ This is likely an underestimation as improved diagnostic tools nowadays enable us to diagnose many more strokes that would have gone undetected previously. There is virtually no documented data concerning functionality and independence in post-stroke Saudi patients. Data regarding the prevalence of kidney dysfunction is better documented with the introduction of routine renal function screening for diabetics. The ubiquity of type 2 diabetes has made impaired kidney function quite common in the region.^7^ The use of renal biomarkers in predicting the disability and complications of stroke is an area that remains almost completely uninvestigated. In this study, we have attempted to correlate renal dysfunction with stroke complications and outcomes.

## Methods

The study design was a retrospective cohort design. The study population consisted of a cohort of acute stroke patients admitted to King AbdulAziz Medical City in Riyadh, Saudi Arabia in the period between January 2012 until June 2015. Of the 1121 adults admitted, we excluded patients with documented known glomerular diseases. We also excluded patients who had no documented ischemic or hemorrhagic insult on imaging (i.e. transient ischemic attacks) and patients with cerebral venous thromboses. Only adult patients over the age of 18 years were included. After applying the exclusion criteria, a total of 727 patients were eligible.

The charts of the included patients were reviewed. The study protocol was approved by the center’s institutional review board. Consent was exempted as this was a retrospective chart review. Patient confidentiality was maintained.

Data was collected by review of paper and electronic records. Collected information included demographics, laboratory values on admission, diagnoses of stroke type, history of vascular risk factors (including hypertension, diabetes, dyslipidemia, ischemic heart disease, documented atrial fibrillation and history of smoking) as well as data regarding the patients’ neurological status via National Institute of Health Stroke Score (NIHSS) and functional status; modified Rankin Scale (mRS) on admission and on discharge. Hypertension was defined as a systolic blood pressure ≥140, a diastolic blood pressure ≥90 mmHg or use of antihypertensive medications. Diabetes was defined according to the 2015 American Diabetes Association practice recommendations.^8^ Dyslipidemia was defined according to the fourth Adult Treatment Panel guidelines, treatment with a lipid lowering agent or a self-reported history of dyslipidemia.^9^ Atrial fibrillation was defined by at least one electrocardiogram documenting it. Ischemic heart disease (IHD) was defined according to the 2014 AHA/ACC joint guideline, a positive history of percutaneous coronary intervention or bypass graft surgery.^10^

The Modification of Diet in Renal Disease (MDRD) equation was used to estimate GFR. No coefficient was used:^11^ GFR (mL/min/1.73 m^2^) = 175×(Serum creatinine)^-1.154^ × (Age)^-0.203^ × (0.742 if female).

Outcome data included: in-hospital death, functional disability post-stroke [mRS at discharge], degree of neurological deficit [NIHSS at discharge] and occurrence of stroke-related complications including: aspiration pneumonia, urinary tract infections (UTI), venous thromboembolism, stroke recurrence, falls and bedsores. A composite outcome was proposed in order to quantify patients who had died and/or those who had experienced complications.

The eGFR values were classified according to Kidney Disease: Improving Global Outcomes (KDIGO) clinical guideline into: eGFR >60 and eGFR ≤60 mL/min/1.73 m^2^.^11^ This cutoff was chosen to override expected eGFR variations as a level of 60 mL/min/1.73 m^2^ indicates kidney dysfunction that cannot be attributed to age or body mass variations. Categorical variables were expressed as frequencies. Numerical variables were expressed as means and standard deviations. Comparison between the baseline characteristics and outcomes of eGFR groups was conducted using the Pearson chi square test for categorical variables and Student t-test for numerical variables.

Odds ratios (OR) for complications, death and the composite outcome were also calculated. The degree of residual neurological deficit was expressed by NIHSS on discharge. Post-stroke functionality was expressed by mRS at discharge. Stroke severity was categorized according to NIHSS into mild (NIHSS≤5), moderate (NIHSS=6-14) and severe (NIHSS>14).^12^ Disability was classified into mild (mRS≤2) and severe (mRS>2). A binary logistic regression model was applied to assess the independent risk that eGFR contributed to mortality and achieving the composite outcome.

Analysis was conducted using Statistical Package for the Social Sciences version 22.0 (SPSS, IBM Corp., Chicago, IL). The limit of statistical significance was set at *p*<0.05.

## Results

Baseline demographics, functional status and risk factor profiles for patients according to the eGFR categories are presented in **Table 1**. The mean age of the sample was 59.13±13.12. The mean eGFR for the sample was 84.93 mL/min/1.73 m^2^. The overall sample showed an expected higher overall frequency of ischemic strokes versus hemorrhagic strokes. There was no statistical difference in stroke type amongst the 2 groups (*p*=0.349), nor were there significant age (*p*=0.187) or gender differences (*p*=0.878) amongst them.

**Table 1:** Baseline patients characteristics according to eGFR categories.

Characteristics	Estimated GFR (eGFR) Levels		Total n (%)	*P*-value*
	eGFR >60 mL/min/1.73 m^2^	eGFR ≤60 mL/min/1.73 m^2^			
Overall percentage in sample	596 (82.0)	131 (18.0)	727 (100)	
eGFR, mL/min/1.73 m^2^, mean±SD	94.08±19.296	43.27±14.094		
Age, years, mean±SD	58.22±13.07	63.31±12.50		0.187
***Gender***				0.878
Male	409 (68.6)	89 (31.4)	498 (68.5)	
Female	187 (67.9)	42 (32.1)	229 (31.5)	
***Type of stroke***				0.349
Ischemic stroke	523 (87.8)	111 (84.7)	634 (87.2)	
Hemorrhagic stroke	73 (12.2)	20 (15.3)	93 (12.8)	
***Vascular comorbidities***				
Hypertension	481 (80.7)	123 (93.9)	604 (83.1)	0.000
Diabetes	393 (65.9)	107 (81.7)	500 (68.8)	0.000
Dyslipidemia	457 (76.7)	106 (80.9)	563 (77.4)	0.293
History of Ischemic heart disease	95 (15.9)	39 (29.8)	134 (18.4)	0.000
Atrial Fibrillation	46 (7.7)	18 (13.7)	64 (8.8)	0.028
Smoking	106 (17.8)	21 (16.0)	127 (17.5)	0.632
Baseline NIHSS on admission, mean±SD	5.33±5.79	5.69±6.630		0.532
***Baseline NIHSS category^‡^***				0.060
Mild stroke ≤5 (Intact neurologically at baseline)	385 (65.5)	80 (64.5)	465 (65.3)	
Moderate stroke 6-14 (Moderate neurological impairment at baseline)	141 (24.0)	31 (25.0)	172 (24.2)	
Severe stroke >14 (Severe neurological impairment at baseline)	62 (10.5)	13 (10.5)	75 (10.5)	
***Baseline mRS category^†^***				0.010
No disability to mild disability (mRS 0-2)	562 (95.4)	112 (89.6)	674 (94.4)	
Moderate to severe disability (mRS 3-6)	27 (4.6)	13 (10.4)	40 (5.6)	

* *p*-value indicates eGFR> 60 compared with eGFR≤60. T-tests was used to compare means. Chi-square test was used to compare categories,

‡ missing in 15 cases, analysis carried out on 712 cases,

† missing in 13 cases, analysis carried out on 714 cases, mRS - Modified Rankin Scale, NIHSS - National Institutes of Health Stroke Scale Score, eGFR - glomerular filtration rate, Values are expressed as numbers and percentage n (%),

Patients with low eGFR values had a higher proportion of diabetics and hypertensives amongst them. Dyslipidemia was the most prevalent cardiovascular risk factor, occurring in 77.4% of the sample but showed no significant difference when the two eGFR groups were compared (*p*=0.293). The incidence of IHD was doubled in the low eGFR group 29.8% compared to the high eGFR group 15.9% (*p*≤0.001). The overall frequency of atrial fibrillation was also significantly higher for patients with low eGFR (*p*=0.028).

Both groups experienced strokes of similar severity with similar mean NIHSS values at presentation (*p*=0.060). Patients with low eGFR were significantly more likely to be functionally impaired (had mRS> 2 on admission) compared to patients with higher eGFR (*p*=0.010).

Outcomes amongst eGFR groups and adjusted Odds ratios for the low eGFR group are presented in **Table 2**. When all contributing risk factors were controlled for, patients with low eGFRs were almost 4 times more likely to experience all-cause mortality during admission (OR=3.772, 95% CI=1.609–8.844). They also had a significant risk of achieving the composite outcome (OR=1.781; 95% CI=1.153–2.751). Patients with lower eGFR were significantly more likely to experience UTIs while hospitalized at a rate double that of the high eGFR group (OR=2.047, 95% CI=1.024–4.093).

**Table 2:** Outcome measures amongst eGFR groups.

Outcomes	Estimated GFR (eGFR) levels		*p*-value	Adjusted OR	OR, 95% C. I. (Lower – Upper)
	>60 mL/min/1.73 m^2^ (n=596)	≤ 60 mL/min/1.73 m^2^ (n=131)			
Death	17 (2.9)	14 (10.7)	0.002	3.772	(1.609 – 8.844)
Composite outcome	147 (24.7)	56 (42.7)	0.009	1.781	(1.153 – 2.751)
***In hospital complications***					
Pneumonia	37 (6.2)	8 (6.1)	0.733	0.866	(0.380 – 1.976)
UTI	30 (5.0)	17 (13.0)	0.043	2.047	(1.024 – 4.093)
VTE (DVT/PE)	12 (2.0)	1 (0.8)	0.224	0.267	(0.032– 2.243)
Fall	15 (2.5)	1 (0.8)	0.220	0.276	(0.035 – 2.158)
Bedsore	4 (0.7)	5 (3.8)	0.097	3.466	(0.800 – 15.010)
Recurrence of stroke/ TIA	59 (9.9)	22 (16.8)	0.180	1.476	(0.835 – 2.609)
Neurological deterioration	38 (6.4)	14 (10.7)	0.243	1.514	(0.755 – 3.038)
***mRS disability category post-stroke at discharge***					
Mildly disabled (0-2)	406 (72.6)	71 (61.7)	0.010
Moderately to severely disabled (3-6)	153 (27.4)	44 (38.3)	
NIHSS value at discharge, mean±SD	4.18±7.567	7.14±11.882	0.011		
***NIHSS severity category at discharge***			
Mild stroke (≤ 5)	448 (80.1)	83 (72.2)	0.011
Moderate stroke (6 - 14)	63 (11.3)	18 (15.7)	
Severe stroke (>14)	48 (8.6)	14 (12.2)	

eGFR - estimated glomerular filtration rate, UTI - urinary track infection, VTE - venous thromboembolism, DVT - deep venous thrombosis, PE - pulmonary embolism, TIA - transient ischemic attacks, mRS - modified Rankin Scale, NIHSS - National Institute of Health Stroke Score, Values are expressed as numbers and percentage n (%)

The discharge mRS was higher in the group with lower eGFR and these patients were also more likely to have a greater degree of residual neurological deficit with higher NIHSS at discharge meaning they were less likely to return to functional independence after their strokes.

## Discussion

The mean age of the sample was around 59.13 years, which is far below the mean age in other regions.^13^ The average age of stroke in American men was 68.8 and the average age for women was even higher at 72.3 years. The mean age of the sample was slightly younger than reported by a prior study in the same center 20 years ago, which was 63 years.^6^

Hypertension and diabetes were predictably higher in the low eGFR group as both contribute to CKD. The higher proportion of IHD within the low eGFR group is expected due to the role that kidney dysfunction plays in accelerating atherosclerosis.^2^ Patients with eGFR ≤60 were more likely to be disabled and had higher mRS scores on admission. This may be due to them having other limitations of independence associated with CKD. The fact that both groups had similar mean NIHSS at presentation indicates that eGFR levels do not contribute to stroke severity initially.

Patients who died on admission were 3 times more likely to have had low eGFR. The results indicate that kidney dysfunction independently affects the likelihood of post-stroke mortality when controlling for vascular risk factors. Patients who experienced a UTI during their admission were twice more likely to have had low eGFR. The incidence of UTI in CKD is not known to be different from the general population. Studies have postulated that the higher proportion of diabetics within this population may lead to a higher rate of UTIs.^14^ Low eGFR was also found to be associated with higher rates of functional impairment as patients with mRS >2 at discharge were more likely to have had low eGFR (*p*=0.010).

Finally, a higher proportion of post-stroke residual deficits were documented in patients with low eGFRs since a greater proportion of patients within this group were discharged with strokes classified as moderate or severe (*p*=0.011). This is interesting since eGFR was not found to contribute to stroke severity at presentation.

It is worth noting that our study had some limitations. Firstly, our center routinely documents NIHSS and mRS at discharge after generally short admissions. This may not truly reflect the neurological and functional status of resolved stroke. Secondly, we have only assessed for in-hospital outcomes where many of these outcomes may occur post-discharge.

In conclusion, the effect of kidney dysfunction on stroke outcomes is complex and further studies are needed to appreciate this interaction. Our findings establish that there is a role that CKD plays in acute stroke and provide evidence supporting the use of eGFR as a predictor of outcomes.
